# Characterization of Circular RNAs in Chinese Buffalo (*Bubalus* *bubalis*) Adipose Tissue: A Focus on Circular RNAs Involved in Fat Deposition

**DOI:** 10.3390/ani9070403

**Published:** 2019-07-01

**Authors:** Jieping Huang, Jinhui Zhao, Qiuzhi Zheng, Shuzhe Wang, Xuefeng Wei, Fen Li, Jianghua Shang, Chuzhao Lei, Yun Ma

**Affiliations:** 1 College of Life Sciences, Xinyang Normal University, Xinyang 464000, Henan, China; 2 Institute for Conservation and Utilization of Agro-Bioresources in Dabie Mountains, Xinyang, Henan 464000, China; 3 Key Laboratory of Buffalo Genetics, Breeding and Reproduction Technology, Ministry of Agriculture and Guangxi, Buffalo Research Institute, Chinese Academy of Agricultural Sciences, Nanning 530001, China; 4 College of Animal Science and Technology, Northwest A&F University, Yangling 712100, Shaanxi, China

**Keywords:** *Bubalus bubalis*, adipose tissue, RNA sequencing, circular RNA, fat deposition

## Abstract

**Simple Summary:**

Buffalo play a vital role in several southeastern and middle-eastern Asian countries and Africa. Fat deposition has received increased attention because of its importance to meat quality. However, information on the development of buffalo adipose tissue is poorly understood and requires further investigation. This study characterized circular RNA (circRNA) profiles of adipose tissues in different developmental stages in buffalo. Co-expression analysis and functional enrichment revealed a considerable number of circRNAs which may function in fat deposition. Further qRT-PCR analysis identified two circRNAs with a significant association with PR/SET domain 16, a fat metabolism gene. Our results identified candidate regulators of fat deposition in buffalo, which may be valuable for buffalo breeding.

**Abstract:**

Circular RNAs (circRNAs) have been identified as a novel type of regulators involved in multiple biological processes. However, circRNAs with a potential function in fat deposition in buffalo are poorly understood. In this study, six RNA libraries of adipose tissue were constructed for three young and three adult Chinese buffaloes with paired-ends RNA sequencing using the Illumina HiSeq 3000 platform. A total of 5141 circRNAs were computationally identified. Among them, 252 circRNAs were differentially expressed (DE) between the young and adult buffaloes. Of these, 54 were upregulated and 198 were downregulated in the adult group. Eight DE circRNAs were further identified by quantitative real-time-PCR (qRT-PCR) and Sanger sequencing. Co-expression analysis revealed that 34 circRNAs demonstrated a strong correlation with fat deposition-associated genes (|r| > 0.980). Among these, expressional correlation between two circRNAs (19:45387150|45389986 and 21:6969877|69753491) and PR/SET domain 16 was further verified using qRT-PCR, and a strong correlation was revealed (1 > |r| > 0.8). These results strongly suggest that circRNAs 19:45387150|45389986 and 21:6969877|69753491 are potential regulators of buffalo fat deposition. In summary, this study characterized the circRNA profiles of adipose tissues at different stages for the first time and revealed two circRNAs strongly correlated with fat deposition-associated genes, which provided new candidate regulators for fat deposition in buffalo.

## 1. Introduction

Circular RNAs (circRNAs) are well known as a class of non-coding RNAs with a closed loop. More than 40 years ago, viral circRNAs were identified [1]. Subsequently, circRNAs were observed and identified in eukaryotes [2,3,4]. Due to the rapid advancement of RNA sequencing methodologies and bioinformatics, a great number of circRNAs have been discovered in many species in recent years. Originally, circRNAs were considered byproducts generated from an error in alternative splicing [5]. In recent years, circRNAs have been found to be involved in multiple biological processes [6,7,8]. In humans, many tissue-specific circRNAs have been identified [9,10,11], and some of them play roles in disease [12] or can be cancer biomarkers [13,14]. In mice, circRNA_010567 promotes myocardial fibrosis via miR-141 suppression by targeting TGF-β1 [15]. Similarly, circRNA_100782 regulates proliferation of pancreatic carcinoma [16]. Interestingly, expression of circRNAs can be induced by chemicals [17], implicating circRNAs in the response to external injury. In livestock animals, a great number of circRNAs have been identified by RNA sequencing technology, and some of them are associated with economically important traits. Based on RNA sequencing, 13,950 circRNAs have been identified in the preovulatory ovarian follicles of goats [18]. Among these, chi_circ_0008219 is a potential circRNA associated with goat reproductive traits [18]. In swine, 6,130 circRNAs have been identified in the muscle, and most of them are uniquely expressed in one breed [19], suggesting that circRNAs may be involved in maintaining characteristics of a breed. In cattle, 12,918 circRNAs have been identified in the muscle tissue [20]. The circLMO7 is abundant in muscle tissue and regulates myoblast differentiation and survival [20]. Characterizing the circRNA profiles of specific tissues or cells is a promising way to reveal functional circRNAs.

Beef (cattle meat) is popular all over the world for its high protein and vitamin content. Meat tenderness and juiciness are affected by the intramuscular fat (IMF) content. In recent years, cattle meat has been imported to meet consumer demand in China [21]. Although buffalo (*Bubalus bubalis*) is abundant in China, buffalo meat has not been widely accepted by customers because of the low IMF content [22]. Besides, the IMF level is too limited to be sampled for RNA sequencing analysis. Many studies demonstrate that back subcutaneous fat is significantly associated with IMF [23,24], which indicates that the character of backfat tissue may be used as an indicator of IMF. However, development of backfat tissue is poorly understood and requires further investigation in buffalo. Recently, circRNA expression profiles have been characterized in adipose tissues or adipose-associated cells of humans [25], mice [26], and swine [27]. These results provide vital information for further studies on fat deposition. However, to date, there has been no study characterizing the circRNA profiles in buffalo adipose tissue. Adipose tissue is formed at specific times to meet the requirement of an organism’s development. Generally, capacity of fat deposition in adult animals is higher than that of young animals. In the present study, circRNA profiles of subcutaneous adipose tissue of Chinese buffalo at two developmental stages (i.e., young and adult) were identified using paired-end sequencing using the Illumina HiSeq 3000 platform. We aimed to identify circRNAs with a significant association with fat deposition for further research on adipose tissue development in buffalo.

## 2. Materials and Methods

### 2.1. Animal Ethics

All animals were bred for commercial use rather than for experimental reasons, and they were slaughtered according to the food industry-approved halal food quality certified protocol by a Muslim cleric according to the law of Islam. Thus, no ethics approval was required by a specific committee.

### 2.2. Animals and Tissue Samples

For RNA sequencing, six Chinese buffaloes (swamp type) were sampled, including three young (6-month-old) and three adult (30 month old) individuals. In addition, another 49 buffaloes with variable months of age were also sampled for a subsequent validation assay. The buffaloes were raised at the Xinyang buffalo farm (Xinyang, Henan, China) with equivalent forage and feeding management conditions. Subcutaneous adipose was harvested after slaughter and frozen immediately in liquid nitrogen.

### 2.3. RNA Isolation and Sequencing

Total RNA was extracted using TRIzol (Invitrogen, Carlsbad, CA, USA) according to the manufacturer’s instructions. The RNA quantity was verified with the NanoDrop 2000 (Nanodrop, Wilmington, DE, USA) and 1.5% agarose gel. The rRNA was removed by the Epicentre Ribo-zero rRNA Removal Kit (Epicentre, San Diego, CA, USA). The RNA was fragmented, and random primers were added to yield double-stranded cDNA. Then, the A-tail and ligating adapters were added to the cDNA, and PCR was performed to enrich the cDNA. The cDNA was precipitated with ethanol, and its quality was assessed using an Agilent Bioanalyzer 2100 system (Agilent, Santa Clara, CA, USA). Quantification of the sample was performed using the Quant-iT™ PicoGreen^®^ dsDNA Assay Kit (Life, Southfield, MI, USA). Finally, the cDNA library was sequenced using the double terminal sequencing mode of the Illumina HiSeq 3000 platform. The RNA sequencing data were deposited in the NCBI Gene Expression Omnibus (GEO), and the accession number is GSE112744.

### 2.4. Quality Control, Transcriptome Assembly, and CircRNA Prediction

Trim Galore was used to remove adapter sequences and low-quality sequences [28]. The FastQC function in Trim Galore was used for quality control and calculating the proportion of Q20 and Q30 [28]. Reads with high quality were collected and used for analysis.

The cattle genome (UMD3.1) was used as a reference genome because the buffalo genome has not been successfully assembled yet. The cattle reference genome and the gene model annotation files were downloaded from the Ensemble database. High-quality reads for each sample were aligned to the reference genome by STAR [29]. For aligned sequence 6% mismatch was allowed; the maximum total length mapped to genome was 1,000,000 bp; minimum intron length was 20 bp; maximum intron length was 1,000,000 bp; other parameters were set to default values. For circRNA prediction, the CIRCexplorer2 was employed to identify the back-spliced junction of circRNAs [30]. The host genes of circRNAs, excluding the intergenic circRNAs and the circRNAs produced from transcripts without gene symbols, were statistically analyzed.

### 2.5. Differential Expression, Functional Enrichment, and Co-Expression Analysis

To calculate the expression levels of circRNAs, back-spliced reads per million mapped reads (BSRP) was used. To screen for differentially expressed (DE) circRNAs, DESeq2 software (Version 1.14.1) was used [31], and those with a fold-change ≥ 2 and a *p*-value ≤ 0.05 were considered as DE circRNAs. The host genes of DE circRNAs were used for functional enrichment by DAVID [32]. A *p*-value ≤ 0.05 was used as threshold to evaluate significant enrichment. Only items associated with lipid metabolism were retained.

A number of DE genes were previously identified in adipose tissues of 6 and 30 month old buffaloes. Among them, 11 genes (peroxisome proliferator activated receptor gamma (PPARG), serum/glucocorticoid regulated kinase 1 (SGK1), cyclin dependent kinase 5 (CDK5), sterol regulatory element binding transcription factor 1 (SREBF1), thyroid hormone responsive (THRSP), FYN proto-oncogene (FYN), SMAD family member 3 (SMAD3), sterol regulatory element binding transcription factor 2 (SREBF2), secreted frizzled related protein 4 (SFRP4), PR/SET domain 16 (PRDM16), and bone morphogenetic protein 7 (BMP7)) involved in adipogenesis [33,34] were included (Table S1). To search for potential circRNAs involved in fat deposition, co-expression analysis was performed for these 11 DE genes and the DE circRNAs. The 1 > |r| > 0.8 means a strong correlation. To obtain a reliable result, only those mRNA–circRNA pairs with |r| > 0.980 were retained for the small sample size (*n* = 6) used in RNA sequencing.

### 2.6. Validation Assay

Quantitative real-time-PCR (qRT-PCR) and Sanger sequencing were performed for circRNA validation. Primers were designed by the Pick Primers function in NCBI (Table 1). Reverse transcription was performed using the PrimeScript RT Reagent Kit with gDNA Eraser (TaKaRa, Dalian, China). The qRT-PCR was performed to detect the expression levels of circRNAs and mRNAs using SYBR Green I (TaKaRa, Dalian, China) with two-step reactions, as recommended by the manufacturer’s protocol. The *GAPDH* was used as an internal control gene [35]. The cycle threshold (2^−ΔΔCt^) method was used to calculate relative expression levels of circRNA and mRNA. Relative expression levels of each tissue are presented as the means ± SEMs. Meanwhile, the PCR products were sequenced to validate the junctions of circRNAs.

## 3. Results

### 3.1. Overview of Sequencing and Quality Control

**Figure 1:**
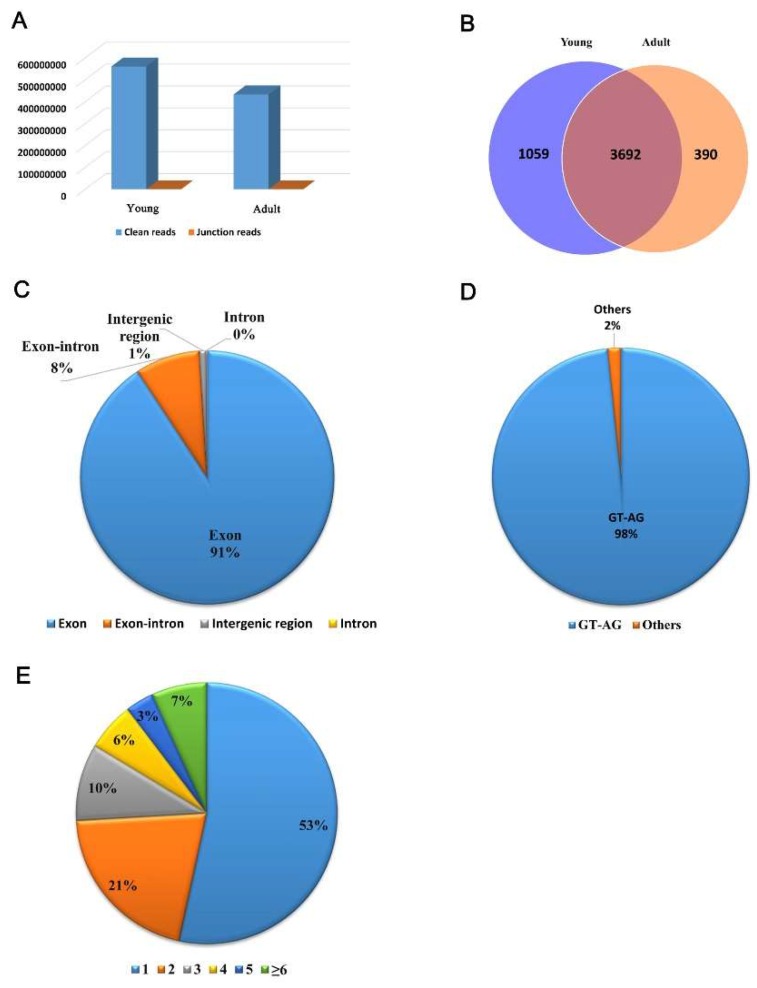
Profiling of circRNAs in young and adult Chinese buffalo adipose tissue. (**A**) Clean reads and junction reads obtained from young and adult buffalo adipose tissue. (**B**) circRNAs expressed in young and adult buffalo adipose tissue. (**C**) Overview of the types of total predicted circRNAs. (**D**) Splicing modes at the junctions. (**E**) Distribution of host genes producing different numbers of circRNAs. Different colors mean different numbers of circRNAs produced by the same host gene.

A total of six cDNA libraries of adipose tissues were constructed for RNA sequencing, including three young and three adult buffaloes. There were 561 million and 433 million clean reads obtained from young and adult buffaloes, respectively (Table S2). Since the buffalo genome assembly is not available, the cattle genome (UMD3.1) was used as the reference genome. An average of 80.96% of the clean reads were mapped to the reference genome (Table S2). Approximately 0.01% of the clean reads were identified as the junction reads for each sample (Figure 1A).

### 3.2. CircRNAs Expressed in Back Subcutaneous Adipose Tissue of Buffalo

In total, 5141 circRNAs were computationally identified in the present study (Table S3). Among them, 501 circRNAs have been previously published for cattle [20], and the remainder were considered as novel bovine circRNAs. There were more circRNAs specifically expressed in the young group (1059) than that in the adult group (390), with 2,392 circRNAs being expressed in both developmental stages (Figure 1B). Based on the junction positions, the identified circRNAs were divided into four types: exon circRNA, exon–intron circRNA, intergenic circRNA, and intron circRNA. Most of the circRNAs identified were exon type (4,665, 90.74%), followed by exon–intron type (425, 8.27%), intergenic type (45, 0.88%), and intron type (6, 0.12%) (Figure 1C). Most of the junctions were flanked by the classical GT-AG splicing sites (98.40%, Figure 1D).

The circRNAs were produced from 2,187 host genes. A host gene could produce multiple circRNAs (Table S3, Figure 1E). About half of the host genes (1168, 53.40%) produced only a circRNA. Approximately 7% of host genes produced more than six circRNAs. The maximum number of circRNAs produced from a host gene was 32.

### 3.3. Identification of DE circRNAs and Functional Enrichment

Of the 5141 circRNAs, a total of 252 DE (*p* < 0.01) circRNAs were identified between the young and adult groups, including 54 upregulated (21.43%) and 198 downregulated (78.57%) circRNAs in adult buffaloes (Table S4). Among them, 20 circRNAs were downregulated with an absolute fold-change ≥ 4, and the maximum absolute fold-change was 6.18 (Table S4). However, all the upregulated circRNAs demonstrated a fold-change less than 4. Most of the DE circRNAs were exon type, and there was no intron circRNA, indicating that circRNAs produced from exons might play a more important role in the development of buffalo adipose tissue. Host genes of DE circRNAs were used for functional enrichment. The primary aim of this study was to identify circRNAs implicated in fat deposition. However, no term associated with lipid metabolism was found (data not shown).

### 3.4. Co-Expression Analysis Revealed circRNAs Associated with Fat Deposition

While the functional enrichment of the host genes did not detect any associations between specific circRNAs and fat deposition, the co-expression analysis revealed a total of 37 circRNA–mRNA pairs had an |r| > 0.980 (Table 2), including 10 pairs with a negative correlation and 27 pairs with a positive correlation. A total of 34 circRNAs and six mRNAs (*CDK5*, *PPARG*, *PRDM16*, *SFRP4*, *SREBF2*, and *THRSP*) were involved.

### 3.5. Validation of DE circRNAs and circRNA–mRNA Pairs

**Figure 2:**
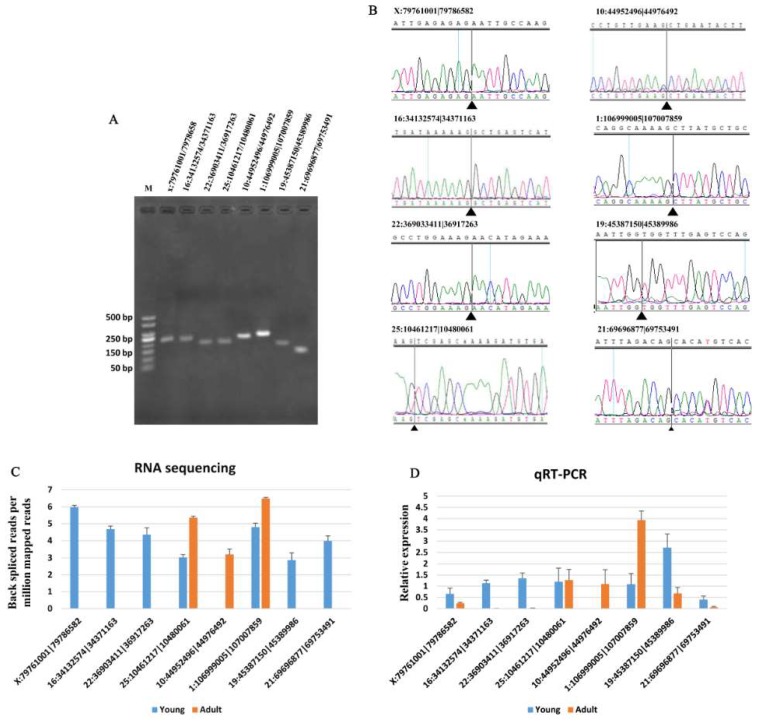
Validation of eight DE circRNA expression levels. (**A**) Electrophoretogram of the qRT-PCR products of the eight circRNAs obtained with divergent primers. M: marker 500. (**B**) Sanger sequencing of the eight circRNAs confirms the head-to-tail splicing. (**C**) Expression pattern of the eight circRNAs between young and adult Chinese buffalo adipose tissue based on RNA sequencing. (**D**) Validation of the expression pattern for the eight DE circRNAs by qRT-PCR. All of the eight circRNAs confirmed the RNA sequencing pattern. Data are presented as the means ± SEMs, *n* = 3.

To evaluate the reliability of the DE analysis results, eight DE circRNAs (Table 1) were selected randomly for validation by qRT-PCR. Fragments containing the junctions of circRNAs were clearly amplified (Figure 2A). The fragment sequences obtained using Sanger sequencing further confirmed the back-splice junctions (Figure 2B). The expression patterns of the eight circRNAs were consistent with those obtained from RNA sequencing (Figure 2C,D).

**Figure 3:**
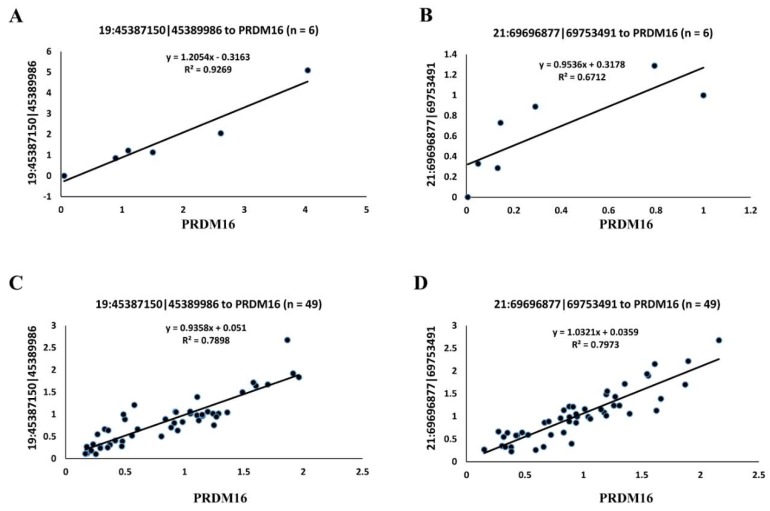
Validation of the two circRNAs with a strong correlation to *PRDM16* by qRT-PCR. The *y*-axis shows the relative expression of circRNA and *PRDM16*. Pearson’s correlation coefficients between circRNA and PRDM16 based on their relative expression were calculated to yield an *r*-value. (**A**) Validation for 19:45387150|45389986-PRDM16 in young and adult buffalo, *n* = 6. (**B**) Validation for 21:6969877|69753491-PRDM16 in young and adult buffalo, *n* = 6. (**C**) Validation for 19:45387150|45389986-PRDM16 in buffalo with variable months of age, *n* = 49. (**D**) Validation for 21:6969877|69753491-PRDM16 in buffalo with variable months of age, *n* = 49.

In addition, the expressional correlation of two circRNAs (19:45387150|45389986 and 21:6969877|69753491) paired with the *PRDM16* transcript were confirmed by qRT-PCR as well. First, a validation assay was performed using adipose tissues of three young and three adult buffaloes. In this part, r values for 19:45387150|45389986-PRDM16 and 21:6969877|69753491-PRDM16 were 0.963 and 0.893, respectively (Figure 3A,B, Table 3). Then, adipose tissue samples from another 49 buffaloes with variable months of age were further used for validation, and the r values for 19:45387150|45389986-PRDM16 and 21:6969877|69753491-PRDM16 were 0.889 and 0.893, respectively (Figure 3C,D, Table 3).

## 4. Discussion

To identify circRNAs with a potential function in fat deposition in buffalo, circRNA expression profiles of adipose tissues were characterized by Ribo-Zero ribonucleic acid sequencing in Chinese buffalo. The RNA library construction by removing rRNAs and linear RNAs is more effective for circRNA identification [36]. In the present study, the RNA library was constructed and 5141 circRNAs were identified which was comparable with previous studies constructing libraries by removing rRNAs and linear RNAs [18,20]. Most of the circRNAs identified were exon type (Figure 1C), and ~98% of junctions reflected the classical GT-AG splicing mode (Figure 1D). In addition, 501 of the 5141 circRNAs were previously identified in the muscle tissue of Qinchuan cattle [20]. Our results further demonstrate that constructing an rRNA-depleted library without removing linear RNAs is practical for circRNA identification. Differential expression analysis revealed 252 DE circRNAs between the young and adult groups (Table S4). The validation results indicate a high reliability of the circRNA prediction.

For genes, a specific expression profile indicates a specific role and function. The circRNAs were expressed more abundantly in young buffalo than in adult buffalo (Figure 1B). Meanwhile, most of the DE circRNAs (198/252) were downregulated in adult buffalo (Table S4). According to the mammalian development pattern, a young animal is less efficient at fat accumulation than an old one. Thus, the richer circRNA set in young buffalo indicates that circRNAs may mainly act as negative regulators of fat deposition in Chinese buffalo.

The primary aim of this study was to reveal circRNAs associated with fat deposition. Previous studies have suggested that the host genes of circRNAs can reveal important information on the function of circRNAs [30]. In this study, functional enrichment analysis for the host genes was performed. However, no term associated with fat deposition was identified (*p* < 0.05). In fact, circRNAs participate in biological processes with multiple modes of action [20,37,38], not limited to regulation of their host genes. Therefore, functional enrichment based on the host genes may not be enough. Besides, DAVID is a tool that systematically maps a large number of interesting genes to the associated biological annotation, and statistically highlights the most enriched biological annotation out of thousands of linked terms and contents [39]. However, some genes without strong neighbors will be omitted from the analysis. The limited annotation information of bovines may affect the result as well. Instead, co-expression analysis was used and 34 circRNAs with putative association with fat deposition were screened. Though without systematic and integrative analysis as is available in DAVID, co-expression analysis can directly capture a pair of transcripts with high correlation and should make the study of regulatory mechanisms of circRNAs more difficult.

Initially, 10 of the 37 circRNA–mRNA pairs were selected for validation by qRT-PCR. However, specific primers were available for only three pairs, and high correlations were validated for only two pairs, namely, 19:45387150|45389986-PRDM16 and 21:6969877|69753491-PRDM16. Previous studies have indicated that circRNAs can regulate the expression of their host genes [7,37,40]. The circRNAs 19:45387150|45389986 and 21:6969877|69753491 are produced by N-myristoyltransferase 1 (NMT1) and microtubule affinity regulating kinase 3 (MARK3), respectively (Table 3). N-myristoyltransferase is a key cellular enzyme that carries out lipid modification by facilitating the attachment of myristate (a 14-carbon saturated fatty acid) to the N-terminal glycine of several protein molecules and increases protein lipophilicity [41]. MARK3 is a vital regulator involved in energy metabolism. The *Mark3^-/-^* mice are protected against high-fat diet-induced obesity and display attenuated weight gain [42]. These lines of evidence further suggest that circRNAs 19:45387150|45389986 and 21:6969877|69753491 are likely to be involved in lipid metabolism.

**Figure 4:**
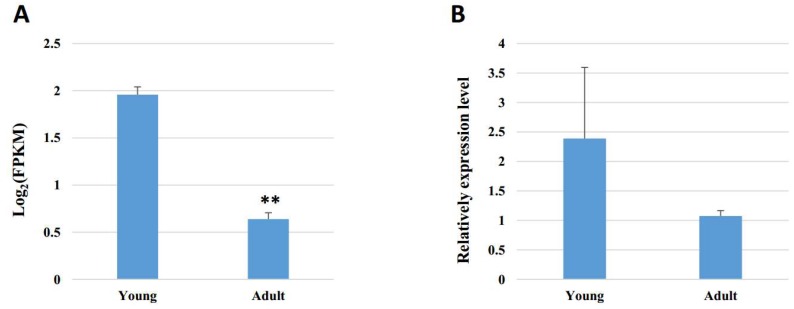
Expression of *PRDM16* in young and adult buffalo adipose tissue. (**A**) Expression pattern analyzed by RNA sequencing. (**B**) Expression pattern analyzed by qRT-PCR. Data are presented as the means ± SEMs, *n* = 3. ** *p* < 0.01, compared with the young group.

In addition, *PRDM16* is identified as a highly expressed gene in brown fat tissue and drives a brown fat-selective transcriptional program [43,44]. Brown adipose tissue can dissipate chemical energy in the form of heat (non-shivering thermogenesis) when encountering cold exposure and excessive feeding, which is considered to exert negative effects on feeding efficiency and inhibit fat deposition in animals [45,46]. In our study, *PRDM16* is richer in young buffaloes than in adult individuals (Table S1, Figure 4). These results indicate that brown adipose tissue is more active in young individuals, which is consistent with the rule of fat development in buffaloes. Both 9:45387150|45389986 and 21:6969877|69753491 demonstrated a positive correlation with *PRDM16*, which indicates that they may inhibit fat deposition in buffalo. However, further functional identification is needed.

## 5. Conclusions

This study characterized the circRNA expression profiles in adipose tissues of young and adult Chinese buffaloes for the first time. We primarily focused on circRNAs with a potential function in fat deposition. Two circRNAs showing a strong correlation with *PRDM16* are likely to inhibit the deposition of white adipose tissue in buffalo. These results provide new candidate factors for further studies of the molecular regulatory mechanism of fat deposition in buffalo.

## Figures and Tables

**Table 1:** Primers used for the validation of differentially expressed circular RNAs (circRNAs) and circRNA–mRNA pairs.

CircRNA ^1^/mRNA	Forward primer (5′–3′)	Reverse primer (5′–3′)	Product Length (bp)
X:79761001|79786582	GCTAAGAACAGAAACACAAAATGCT	CCTCATCTCCAGGGTTTTCTTCA	225
16:34132574|34371163	TCGAACACTCTCTTCAGATGCAA	TGGGGTTTGGATTCTCTGCT	213
22:36903411|36917263	CAGACGGCTCTGGTGCAATA	GTCATCAGCCAGGCTACTCC	180
25:10461217|10480061	AAGGAGTGGGACCTAAAGCC	GGCATTGAAGCGGAAGAAGTC	183
10:44952496|44976492	AACACAGACCTACCGCATCC	GAAAGAGGGCGTAGGTGTCA	234
1:106999005|107007859	CCAGATGTGTGGAGATTGCC	TCCTGGTGGCTGGAAATACC	227
21:69696877|69753491	ATGCAAGTGGAGGTGAAGTGT	TAACTTCTTGCCGCCCATCT	117
19:45387150|45389986	TGGAAGAGGCTAGCAAACGA	GGGCTGAATCTGTCTCGCTG	152
*PRDM16*	TACAGGGTGGAGAAGCGGAA	GTACCTGCACGTGTATCGCT	264
*GAPDH*	CACTCACTCTTCTACCTT	GCCAAATTCATTGTCGTA	91

**^1^** Indicates chromosomal locations in cattle.

**Table 2:** The circRNA–mRNA pairs with potential effects on fat deposition.

Number	circRNA_ID ^1^	Symbol	r Value	Number	circRNA_ID ^1^	Symbol	r-Value
1	10:11563837|11593862	*SREBF2*	−0.9975	20	12:52715274|52733582	*PRDM16*	0.9834
2	15:83640019|83640860	*THRSP*	−0.9960	21	15:22884720|22887938	*SREBF2*	0.9841
3	12:89500403|89506932	*PPARG*	−0.9899	22	12:89492189|89525269	*PRDM16*	0.9841
4	2:37078234|37133108	*THRSP*	−0.9889	23	22:30718181|30723897	*PRDM16*	0.9843
5	12:23235488|23237718	*THRSP*	−0.9889	24	21:69696877|69753491	*PRDM16*	0.9852
6	12:2298522|2305539	*PPARG*	−0.9859	25	4:9762677|9766001	*PRDM16*	0.9854
7	2:37078234|37133108	*CDK5*	−0.9855	26	21:42001223|42024196	*PRDM16*	0.9855
8	12:23235488|23237718	*CDK5*	−0.9855	27	10:60619225|60625573	*SFRP4*	0.9861
9	15:83640019|83640860	*CDK5*	−0.9827	28	11:22513792|22530506	*PRDM16*	0.9865
10	9:84303407|84311225	*THRSP*	−0.9807	29	16:30873113|30903109	*PRDM16*	0.9867
11	X:79782632|79786582	*PRDM16*	0.9805	30	7:18298331|18317388	*SFRP4*	0.9903
12	17:40606888|40608971	*PRDM16*	0.9805	31	14:22698555|22703735	*SFRP4*	0.9906
13	14:28794206|28825387	*PRDM16*	0.9810	32	14:45838481|45839369	*SFRP4*	0.9928
14	11:100361051|100374772	*PRDM16*	0.9814	33	10:70853226|70859881	*PRDM16*	0.9933
15	X:79761001|79786582	*PRDM16*	0.9818	34	2:72283596|72308351	*THRSP*	0.9944
16	17:55314422|55316845	*SFRP4*	0.9828	35	2:37061458|37078299	*PRDM16*	0.9944
17	20:58640529|58642071	*SFRP4*	0.9828	36	5:10860435|10879727	*THRSP*	0.9948
18	9:15487323|15505719	*PRDM16*	0.9833	37	2:72283596|72308351	*CDK5*	0.9955
19	8:91848002|91864189	*PRDM16*	0.9834				

**^1^** Indicates chromosomal locations in cattle.

**Table 3:** Two circRNA–mRNA pairs with potential effects on fat deposition.

CircRNA ^1^	CircRNA Type	Host Gene	mRNA	*r*-Value by RNA-Seq	*r*-Value by qRT-PCR	
				Young and Adult (*n* = 6)	Young and Adult (*n* = 6)	Variable Months of Age (*n* = 49)
19:45387150|45389986	Exon	*NMT1*	PRDM16	0.968	0.963	0.889
21:69696877|69753491	Exon	*MARK3*	PRDM16	0.985	0.893	0.893

**^1^** Indicates chromosomal locations in cattle.

## Supplementary Materials

The following are available online at https://www.mdpi.com/2076-2615/9/7/403/s1, Table S1: Eleven genes (mRNAs) associated with fat deposition showing DE between young and adult buffalo adipose tissues, Table S2: Details of quality control and mapping of RNA sequencing, Table S3: circRNAs identified in adipose tissue of Chinese buffalo based on RNA sequencing, Table S4: DE circRNAs between young and adult buffalo adipose tissue.

## References

[1] Sanger H.L., Klotz G., Riesner D., Gross H.J., Kleinschmidt A.K. (1976). Viroids are single-stranded covalently closed circular RNA molecules existing as highly base-paired rod-like structures. PNAS.

[2] Hsu M.T., Coca-Prados M. (1993). Electron microscopic evidence for the circular form of RNA in the cytoplasm of eukaryotic cells. Nature.

[3] Cocquerelle C., Mascrez B., Hetuin D., Bailleul B. (1993). Mis-splicing yields circular RNA molecules. FASEB J..

[4] Saad F.A., Vitiello L., Merlini L., Mostacciuolo M.L., Oliviero S., Danieli G.A. (1992). A 3′ consensus splice mutation in the human dystrophin gene detected by a screening for intra-exonic deletions. Hum. Mol. Genet..

[5] Cocquerelle C., Daubersies P., Majérus M.A., Kerckaert J.P., Bailleul B. (1992). Splicing with inverted order of exons occurs proximal to large introns. EMBO J..

[6] Memczak S., Jens M., Elefsinioti A., Torti F., Krueger J., Rybak A., Maier L., Mackowiak S.D., Gregersen L.H., Munschauer M. (2014). Circular RNAs are a large class of animal RNAs with regulatory potency. Nature.

[7] Li F., Zhang L., Li W., Deng J., Zheng J., An M., Lu J., Zhou Y. (2015). Circular RNA ITCH has inhibitory effect on ESCC by suppressing the Wnt/β-catenin pathway. Oncotarget.

[8] Liu L., Wang J., Khanabdali R., Kalionis B., Tai X., Xia S. (2017). Circular RNAs: Isolation, characterization and their potential role in diseases. RNA Biol..

[9] Szabo L., Morey R., Palpant N.J., Wang P.L., Afari N., Jiang C., Parast M.M., Murry C.E., Laurent L.C., Salzman J. (2015). Statistically based splicing detection reveals neural enrichment and tissue-specific induction of circular RNA during human fetal development. Genome Biol..

[10] Tan W.L., Lim B.T., Anene-Nzelu C.G., Ackers-Johnson M., Dashi A., See K., Tiang Z., Lee D.P., Chua W.W., Luu T.D. (2017). A landscape of circular RNA expression in the human heart. Cardiovasc. Res..

[11] Xia S., Feng J., Lei L., Hu J., Xia L., Wang J., Xiang Y., Liu L., Zhong S., Han L. (2017). Comprehensive characterization of tissue-specific circular RNAs in the human and mouse genomes. Brief Bioinform..

[12] Lukiw W.J. (2013). Circular RNA (circRNA) in Alzheimer’s disease AD. Front Genet..

[13] Xie H., Ren X., Xin S., Lan X., Lu G., Lin Y., Yang S., Zeng Z., Liao W., Ding Y.Q. (2016). Emerging roles of circRNA_001569 targeting mir-145 in the proliferation and invasion of colorectal cancer. Oncotarget.

[14] Meng S., Zhou H., Feng Z., Xu Z., Tang Y., Li P., Wu M. (2017). CircRNA: Functions and properties of a novel potential biomarker for cancer. Mol. Cancer.

[15] Zhou B., Yu J.W. (2017). A novel identified circular RNA, circRNA_010567, promotes myocardial fibrosis via suppressing miR-141 by targeting TGF-β1. Biochem. Biophysiol. Res. Commun..

[16] Chen G.W., Shi Y.T., Zhang Y., Sun J. (2017). CircRNA_100782 regulates pancreatic carcinoma proliferation through the il6-stat3 pathway. Oncotarg. Ther..

[17] Pei W., Tao L., Zhang L.W., Zhang S., Cao J., Jiao Y., Tong J., Nie J. (2017). Circular RNA profiles in mouse lung tissue induced by radon. Environ. Health Prev..

[18] Tao H., Xiong Q., Zhang F., Zhang N., Liu Y., Suo X., Li X., Yang Q., Chen M. (2017). Circular RNA profiling reveals chi_circ_0008219 function as microRNA sponges in pre-ovulatory ovarian follicles of goats (Capra hircus). Genomics.

[19] Sun J., Xie M., Huang Z., Li H., Chen T., Sun R., Wang J., Xi Q., Wu T., Zhang Y. (2017). Integrated analysis of non-coding RNA and mRNA expression profiles of 2 pig breeds differing in muscle traits. J. Anim. Sci..

[20] Wei X., Li H., Yang J., Hao D., Dong D., Huang Y., Lan X., Plath M., Lei C., Lin F. (2017). Circular RNA profiling reveals an abundant circLMO7 that regulates myoblasts differentiation and survival by sponging miR-378a-3p. Cell Death Dis..

[21] FAO. http://www.fao.org/faostat/zh/#data/TP/visualize.

[22] Tao L., Li J., Zhang Y., Tang S., Huang A. (2014). Study on the meat quality of buffalo in Dehong. Food Ind..

[23] Newcom D.W., Baas T.J., Schwab C.R., Stalder K.J. (2005). Genetic and phenotypic relationships between individual subcutaneous backfat layers and percentage of longissimus intramuscular fat in Duroc swine. J. Anim. Sci..

[24] Jacyno E., Pietruszka A., Kawęcka M., Biel W., Kołodziej-Skalska A. (2015). Phenotypic correlations of backfat thickness with meatiness traits, intramuscular fat, longissimus muscle cholesterol and fatty acid composition in pigs. S. Afr. J. Anim. Sci..

[25] Zhang C., Yin R., Sheng Y., Yang C., He X., Xue W., Huang K. (2018). Comprehensive analysis of the characteristics and differences in adult and newborn brown adipose tissue. Diabetes.

[26] Long T., Guo Z., Han L., Yuan X., Liu L., Jing W., Tian W., Zheng X., Tang W., Long J. (2018). Differential expression profiles of circular RNAs during osteogenic differentiation of mouse adipose-derived stromal cells. Calcif. Tissue Int..

[27] Li A., Huang W., Zhang X., Xie L., Miao X. (2018). Identification and characterization of circRNAs of two pig breeds as a new biomarker in metabolism-related diseases. Cell Physiol. Biochem..

[28] Krueger F. Trim Galore!: A Wrapper Tool around Cutadapt and FastQC to Consistently Apply Quality and Adapter Trimming to FastQ Files. http://www.bioinformatics.babraham.ac.uk/projects/trim_galore/.

[29] Dobin A., Davis C.A., Schlesinger F., Drenkow J., Zaleski C., Jha S., Batut P., Chaisson M., Gingeras T.R. (2013). STAR: Ultrafast universal RNA-seq aligner. Bioinformatics.

[30] Zhang X.O., Dong R., Zhang Y., Zhang J.L., Luo Z., Zhang J., Chen L.L., Yang L. (2016). Diverse alternative back-splicing and alternative splicing landscape of circular RNAs. Genome Res..

[31] Love M., Simon A., Huber W. (2014). Differential analysis of count data-the DESeq2 package. Genome Biol.

[32] DAVID. http://david.abcc.ncifcrf.gov/.

[33] De Sá P.M., Richard A.J., Hang H., Stephens J.M. (2017). Transcriptional regulation of adipogenesis. Comp. Physiol..

[34] Schering L., Albrecht E., Komolka K., Kühn C., Maak S. (2017). Increased expression of thyroid hormone responsive protein (THRSP) is the result but not the cause of higher intramuscular fat content in cattle. Int. J. Biol. Sci..

[35] Li M., Sun X., Cai H., Sun Y., Plath M., Li C., Lan X., Lei C., Lin F., Bai Y. (2016). Long non-coding RNA ADNCR suppresses adipogenic differentiation by targeting miR-204. BBA Gene Regul. Mech..

[36] Chaabane M., Rouchka E.C., Park J.W. Circular RNA Detection from High-throughput Sequencing. Proceedings of the International Conference ACM.

[37] Qu S., Yang X., Li X., Wang J., Gao Y., Shang R., Sun W., Dou K., Li H. (2015). Circular RNA: A new star of noncoding RNAs. Cancer Lett..

[38] Legnini I., Di Timoteo G., Rossi F., Morlando M., Briganti F., Sthandier O., Fatica A., Santini T., Andronache A., Wade M. (2017). Circ-ZNF609 is a circular RNA that can be translated and functions in myogenesis. Mol. Cell.

[39] Huang D.W., Sherman B.T., Lempicki R.A. (2009). Systematic and integrative analysis of large gene lists using DAVID bioinformatics resources. Nat. Protoc..

[40] Zhang Y., Zhang X.O., Chen T., Xiang J.F., Yin Q.F., Xing Y.H., Zhu S., Yang L., Chen L.L. (2013). Circular intronic long noncoding RNAs. Mol. Cell.

[41] Wright M.H., Heal W.P., Mann D.J., Tate E.W. (2010). Protein myristoylation in health and disease. J. Chem. Biol..

[42] Lennerz J.K., Hurov J.B., White L.S., Lewandowski K.T., Prior J.L., Planer G.J., Gereau R.W., Piwnica-Worms D., Schmidt R.E., Piwnica-Worms H. (2010). Loss of Par-1a/MARK3/C-TAK1 kinase leads to reduced adiposity, resistance to hepatic steatosis, and defective gluconeogenesis. Mol. Cell. Biol..

[43] Seale P., Kajimura S., Yang W., Chin S., Rohas L.M., Uldry M., Tavernier G., Langin D., Spiegelman B.M. (2007). Transcriptional control of brown fat determination by PRDM16. Cell Metab..

[44] Kajimura S., Seale P., Tomaru T., Erdjument-Bromage H., Cooper M.P., Ruas J.L., Chin S., Tempst P., Lazar M.A., Spiegelman B.M. (2008). Regulation of the brown and white fat gene programs through a PRDM16/CtBP transcriptional complex. Genes Dev..

[45] Asano H., Yamada T., Hashimoto O., Umemoto T., Sato R., Ohwatari S., Kanamori Y., Terachi T., Funaba M., Matsui T. (2013). Diet-induced changes in Ucp1 expression in bovine adipose tissues. Gen. Comp. Endocrinol..

[46] Komolka K., Albrecht E., Gotoh T., Maak S. (2017). Abundance of beige and brown adipocyte markers in different adipose depots of cattle at 26 months of age. Adv. Anim. Biosci..

